# *Arthroderma tuberculatum* and *Arthroderma multifidum* Isolated from Soils in Rook (*Corvus frugilegus*) Colonies as Producers of Keratinolytic Enzymes and Mineral Forms of N and S

**DOI:** 10.3390/ijerph17249162

**Published:** 2020-12-08

**Authors:** Justyna Bohacz, Michał Możejko, Ignacy Kitowski

**Affiliations:** 1 Department of Environmental Microbiology, Faculty of Agrobioengineering, University of Life Sciences in Lublin, Leszczyńskiego 7 Street, 20-069 Lublin, Poland; michaltomasz.mozejko@gmail.com; 2 State School of Higher Education in Chełm, Institute of Agricultural Science, Pocztowa 54 Street, 22-100 Chełm, Poland; ignacyk@autograf.pl

**Keywords:** *Arthroderma* sp., *Corvus frugilegus*, feathers, keratinases, proteolytic activity, ammonium ions, sulfate ions, keratin hydrolysates, amino acid

## Abstract

Keratinolytic fungi representing the genus *Arthroderma* that were isolated from the soils of a rook (*Corvus frugilegus*) colony were used as biological agents for the disposal of waste feathers. The aim of this study was to assess the abilities of *Arthroderma tuberculatum* and *Arthroderma multifidum* fungi with a varied inflow of keratin matter to biodegrade waste feathers. The evaluation was based on the determination of feather mass loss, the activity of keratinolytic enzymes, and the content of mineral N and S forms. It was found that the activity of protease released by the fungi contributed to an increase in the level of soluble proteins and peptides and the concentration of ammonium ions, as well as alkalization of the culture medium. Keratinase activity was significantly correlated with sulfate release, especially in *A. tuberculatum* cultures. The strains of *A. tuberculatum* fungi isolated from the soil with the highest supply of organic matter, i.e., strains III, IV, and V, had the lowest enzymatic activity, compared to the *A. multifidum* strains, but they released mineral nitrogen and sulfur forms that are highly important for fertilization, as well as nutritionally important peptides and amino acids. *A. tuberculatum* strains can be used for the management of waste feathers that can be applied in agricultural practice.

## 1. Introduction

As reported by Mokrejs et al. [1], between 0.9 and 1.5 billion kilograms of waste feathers are produced annually in the poultry processing industry in the USA. Korniłłowicz-Kowalska and Bohacz [2] and Staroń et al. [3] have reported that the level of the generation of this waste in Poland ranges from approximately 77,000 to 90,000 tons. Globally, this value reaches 2 million tons per year, as shown by Verma et al. [4]. Similar to other keratin waste (hair, bristle, horns, hooves, etc.), chicken feathers contain keratin, i.e., a protein that is characterized by high mechanical strength and resistance to physicochemical factors and the enzymatic lysis associated with the presence of numerous disulfide bridges [5].

Keratin contains high levels of organic N (15–18%) and organic S (5–8%) [6]; therefore, it is a valuable material for industrial use. Chicken feathers serve as an enhancer in the production of biogas [6], fertilizers, feed, and bioactive peptides. They are also used in the cosmetic, medical, and textile industries [7,8,9].

As shown by Mazotto et al. [10], the disposal and recycling of keratin waste, e.g., chicken feathers, is a huge environmental problem. During storage, large amounts of poisonous gases are emitted, i.e., ammonia and hydrogen sulfide, through the uncontrolled microbiological decomposition of the waste [11]. The methods for recycling such material into feed, fertilizers, adhesives, etc. are environmentally unfriendly due to the necessity to use acidic or alkaline chemicals to hydrolyze this protein. Such treatments also require large amounts of energy, which is not only economically disadvantageous but also contributes to the decomposition of nutritionally important essential amino acids, as demonstrated by Cascarosa et al. [12].

The search for environmentally friendly methods for the disposal of this waste is therefore not only a necessity but also an obligation. Methods based on the use of microorganisms, e.g., bacteria, actinomycetes, and fungi, are regarded as environmentally friendly by many researchers [2,13]. Tamreihao et al. [13] emphasized the role of mineral and organic products that are generated in the process of the microbiological decomposition of feathers, which can be used in agriculture. Feather hydrolysates have been proposed for use as nitrogen fertilizers and plant growth stimulants since they are a source of precursors for the plant phytohormone indole-3-acetic acid (IAA).

Keratinolytic fungi are present in environments with a constant flow of organic matter [2]. They are isolated from arable soils [5,14,15,16], sewage sludge and river bottom sediments [17,18], compost [12], bird feathers [19], the hair of free-living rodents [20], the nests of water birds [21], and pellets of birds of prey [22]. They are classified as dermatophytes and fungi representing the Chrysosporium group. Fungi with the ability to decompose keratin have been classified into two orders: Onygenales and Eurotilaes by Błyskal [23]. In agreement with Kunert [24], the author emphasized that Onygenales fungi are highly specialized in keratin degradation. This group includes fungi from the genera *Arthroderma*, *Aphanoascus*, *Epidermophyton*, *Microsporum*, *Trichophyton*, and *Chrysosporium*. The search for species with high keratinolytic activity does not only involve fungi of the genus *Chrysosporium* and the fungi mentioned above [5,25]. Species from the genera *Trichoderma*, *Fusarium, Cladosporium*, *Phytophtora* [26], and *Talaromyces* [27], as well as *Aspergillus niger* and *Aspergillus fumigatus* [19], have been described in the literature as keratin decomposers.

As reported by Bohacz and Korniłłowicz-Kowalska [28], the ability of microorganisms to decompose complex organic matter is stimulated by its presence in the microbial growth environment. Therefore, the isolation of fungi from environments with a high concentration of keratin matter may influence the microbial ability to decompose keratin. An example of such an environment is soil with a constant supply of organic matter from the nests of birds, especially those with a large nesting range, such as the rook *Corvus frugilegus*. This species forms nesting colonies on trees. Due to the loss of feathers by adult birds (molting) in spring and summer [29], very large loads of keratin, as well as minerals originating from droppings and other materials, e.g., food remains, are accumulated in a very small area of soil under trees [30]. Moreover, fallen adults and poorly feathered chicks are very often found in the colonies; hence, a specific microbiome with specific properties is formed in such soils.

Bearing in mind that the use of poultry industry by-products with the involvement of effective microorganisms promotes waste management and brings environmental benefits (limits storage requirements and reduces the release of toxic gases into the atmosphere during uncontrolled decomposition) and that feathers are a source of valuable bioproducts, an attempt was made to use fungi that are effective in the decomposition of waste feathers. The aim of this study was to evaluate the keratinolytic abilities of fungi isolated from soil that was characterized by a constant inflow of keratin matter, i.e., located below nests of the rook *Corvus frugilegus*. To assess the possible effect of microbial growth environment conditions on the specific physiological traits of microorganisms, the research hypothesis assumed that fungi isolated from the immediate range of a rook colony (greater amounts of keratin substrate) have a stronger ability to decompose keratin than those isolated from soil outside the range (lower amounts of keratin substrate).

## 2. Materials and Methods

### 2.1. Isolation of Keratinolytic Fungi

The investigations were carried out on strains of keratinolytic fungi isolated from soils located in two colonies of the rook *Corvus frugilegus* in the villages of Sielec (51°02′24″ N, 23°31′26″ E) and Zagroda (51°01′07″ N, 23°22′19″ E) in Poland. The fungi were isolated using the keratin bait method, with chicken feathers used as a keratin substrate [11,19]. Waste chicken feathers were obtained from the “Superdrob” Poultry Processing Plant in Lublin, Poland. Fungi were isolated from soils in four zones of impact of the rook colonies (zones 1, 2, 3, 4). Zone 1 was an area with a direct colony impact. The establishment of the soil sampling zones was intended not only to demonstrate fungal species diversity in soils with different supplies of keratin matter (unpublished data) but also to show differences in the keratinolytic activity of the fungi, depending on the access of the keratin material. It was assumed that zone 1 would comprise fungi with strong keratinolytic properties, as determined by the presence of the greatest amount of keratin matter.

### 2.2. Identification of Fungal Species

In this study, strains isolated from the soil of zones 1 and 4 were selected for the determination of keratinolytic activity. The species identification of the selected strains was based on an assessment of macroscopic and microscopic features in Petri dishes with Sabouraud medium [31]. Additionally, observations of the mycelium, spores, and fruiting bodies were carried out with the use of an Olympus BX-41 microscope (Olympus, Tokyo, Japan) coupled with a CVIII4 digital camera (Olympus, Tokyo, Japan) integrated with a computer equipped with the Cell-A program (Soft Imaging System GmbH, version (v.) 1.20, Münster, Germany) for analysis, recording, and archiving photos. The species classification based on morphological criteria was completed with the use of specialized systematic keys proposed by Domsch et al. [32] and van Oorschot [33].

PCR and nucleotide sequencing were performed to verify the traditional species identification. DNA was isolated from the mycelium using the CHELEX substrate (Biorad) and enzymes necessary for the digestion of the cell walls, i.e., lyticase (1 mg∙mL^−1^, Sigma-Aldrich, Saint Louis, MO, USA) and proteinase K (20 mg∙mL^−1^, Sigma-Aldrich, Saint Louis, MO, USA). Amplification of the ITS (Internal Transcribed Spacer) fragments using PCR on a DNA template was carried out with the use of specific primers, namely, ITS1: 5-TCCGTAGGTGAACCTGCGG-3 and ITS4: 5-TCCTCCGCTTATTGATATGC-3. The purified PCR products were sequenced with a BigDye Terminator Mix v.3.1 kit (Life Technologies, Thermo Scientific, Waltham, MA, USA), a genetic analyzer ABI3730xl (Life Technologies, Thermo Scientific, Waltham, MA, USA), and the specific primers ITS1-F and ITS4-R (oligo.pl, IBB PAS, Warszawa, Poland). Next, the readings were assembled into appropriate contigs, thus obtaining a consensus sequence. The sequences were compared with the UNITE database (https://unite.ut.ee/) using the BLAST program [34]. Details of the reaction conditions are described elsewhere [12].

### 2.3. Determination of Keratinolytic Activity

Three strains from each of the two species *Arthroderma tuberculatum* Kuehn (teleomorph of *Chryspsorium tuberculatum*) and *Arthroderma multifidum* C.O. Dawson (teleomorph of *Chryspsporium* sp. Corda) were selected for the determination of keratinolytic activity. The *Arthroderma tuberculatum* and *Arthroderma multifidum* strains originated from the zone 1 and zone 4 soils, respectively.

The fungi were cultured at 28 °C on a liquid mineral medium containing 1% of fragmented chicken feathers as a sole source of C, N, and S [5]. The ability of these fungi to biodegrade waste feathers was assessed periodically, i.e., after 4, 8, 12, 16, 20, and 27 days of cultivation. Three replicates were designed for each experimental term. The keratinolytic activity was determined via measurements of the protease activity using the Anson [35] method, which was modified by Korniłłowicz [36] using 1% casein as a substrate in a 0.1 M phosphate buffer at pH 7.8 with the addition of magnesium ions (1 mM MgCl_2_), where measurements of the keratinase activity were undertaken with the methodology proposed by Anbu et al. [37]. The keratinolytic activity was presented as the specific activity (units of activity per milligram of protein), and the proteolytic activity was shown in micrograms of tyrosine per milligram of protein.

### 2.4. Determination of Organic and Mineral Bioproducts of Waste Feather Biodegradation

#### 2.4.1. Mineral Forms of N and S in Post-Culture Fluids

The content of soluble protein and peptides was determined using the Lowry method [38]. The mineral N and S forms were determined as described by Bohacz and Korniłłowicz-Kowalska [12]. The potentiometric method was employed for the determination of the pH value in the post-culture fluids.

#### 2.4.2. The Amino Acid Composition in Post-Culture Fluids

The amounts of the amino acids Asp, Thr, Ser, Glu, Pro, Gly, Ala, Cys, Val, Met, Ile, Leu, Tyr, Phe, His, Lys, Arg, and Trp were determined in the post-culture fluids. The amino acid composition in the *Arthroderma tuberculatum* (III) post-culture fluids was determined with an AAA 400 amino acid analyzer from Ingos (Prague, Czech Republic). They were separated using ion-exchange chromatography. A 0.37 × 45 cm column was filled with ion-exchange resin. LG ANB OSTION (Ingos, Prague, Czech Republic), a cation exchanger with an average grain size of approximately 12 µm in the form of Na, was used for the hydrolysates. The column temperature was set to 60 °C and 74 °C. Ninhydrin (Ingos, Prague, Czech Republic) was used as a reagent for amino acid detection. The amino acids were identified with the use of a photometric detector at a wavelength of 570 nm for all amino acids, except for proline (440 nm). Four citrate-sodium buffers with NaCl and various pH values were used for elution of amino acids: (1) 0.3 M, pH = 2.6; (2) 0.2 M, pH = 3.0; (3) 0.4 M, pH = 4.25; (4) 1.12 M, pH = 7.9. After the amino acid separation, the column was regenerated with 0.2 N NaOH. The retention time (RT) of the amino acids (minutes) in the analysis conditions had the following values: Asp (15.62), Thr (19.32), Ser (20.87), Glu (26.15), Pro (31.18), Gly (35.95), Ala (37.62), Val (44.27), Ile (51.17), Leu (52.55), Tyr (58.32), Phe (59.97), His (65.52), Lys (67.75), Arg (76.33), cyst acid (5.20), sulf met (15.0), and Trp (25.0). Cysteine was oxidized to cysteic acid and methionine to methionine sulfone using performic acid. In order to determine tryptophan, the sample was subjected to alkaline hydrolysis. The determination was carried out at the Central Research Laboratory of the University of Life Sciences in Lublin.

### 2.5. Statistical Analysis

The statistical analyses were carried out using the STATISTICA software v. 13.0 (StatSoft, Kraków, Poland). Principal component analysis (PCA) analysis was performed to demonstrate the correlations between the variables, i.e., protease activity (PA); keratinase activity (KA); the level of soluble proteins and peptides (PR), ammonium (NH_4_), and sulfate (SO_4_) ions; pH of post-culture fluids. The regression functions (fourth-degree polynomial) were determined for the variability of the studied traits among the potentially keratinolytic fungi. To show significant differences between the strains, a one-way analysis of variance (ANOVA) was carried out, followed by a post hoc test with Fisher’s least significant difference (LSD) test at the significance level of α = 0.05.

## 3. Results and Discussion

As reported by Li et al. [39], traditional physicochemical methods for feather disposal are being gradually abandoned due to their negative impact on the environment and the loss of valuable amino acids. Therefore, biological methods based on the use of effective microorganisms are becoming increasingly important.

### 3.1. Identification of Fungi

Based on the macro- and microscopic features, the fungi selected for the analysis were initially classified as the *Ranispora flavissima* species and the genus *Trichophyton* complex (anamorph). The microscopic image showed large globose and verrucose conidia on short side branches and micro- and macroconidia, as well as bone-shaped and spiral cells [32], which indicated the presence of perfect stages.

Based on the PCR reaction and nucleotide sequencing, the fungi were classified as the species *Arthroderma tuberculatum* and *Arthroderma multifidum* (Table 1). As suggested by Oorschot et al. [33], *Arthroderma tuberculatum* and *Arthroderma multifidum* are the perfect stages of *Chrysposroium tuberculatum* and *Chrysosporium* sp. Corda, respectively. The nucleotide sequences of *Arthroderma tuberculatum* were deposited in the GenBank under the following accession numbers: MW 085033, MW 085087, and MW 086602. The following accession numbers were assigned to the nucleotide sequences of *Arthroderma multifidum*: MW 086479, MW 086599, and MW 086601.

### 3.2. Degree of Keratin Substrate Utilization

The ability of the analyzed soil fungi to decompose native keratin was assessed by determining the degree of utilization of native chicken feathers (Table 2). As indicated by Bohacz [5], the loss of feather mass is the most reliable indicator and an “economic coefficient” for evaluation of the keratinolytic abilities of microorganisms. All the *Arthroderma tuberculatum* and *A. multifidum* strains utilized the waste chicken feathers as the sole source of C, N, S, and energy at the level of 30–40%, respectively. Previous investigations conducted by Bohacz [5] demonstrated that the most active keratin decomposers among *Aphanoascus fulvescens* fungi contributed to an approximately 65% loss of keratin substrate weight after 45 days of cultivation. In comparison, actinomycetes represented by *Streptomyces* spp. are capable of 40–90% biodegradation of feathers [39]. As reported by Cãlin et al. [26], fungi of the genera *Trichophyton* spp. and *Chrysoporium* were found to cause a 75% reduction in feather mass on average. The high loss of this keratin substrate may additionally result from the mechanical keratin degradation by means of perforation organs and boring hyphae observed in *Chrysosporium* and *Trichophyton* fungi [2,25]. The statistical analysis of homogeneous groups revealed that the analyzed fungal strains caused a similar level of feather mass loss and did not differ significantly from each other. In turn, significant differences were observed between the *A. tuberculatum* and *A. multifidum* species.

### 3.3. Proteolytic and Keratinolytic Activity

**Figure 1:**
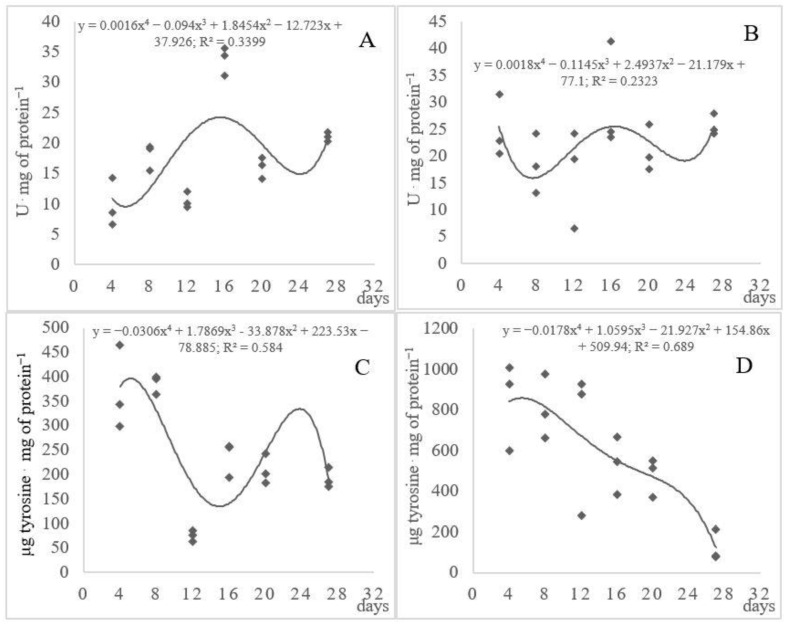
Dynamics of the changes in keratinase (**A**,**B**) and extracellular protease (**C**,**D**) activity in *Arthroderma tuberculatum* (**A**,**C**) and *Arthroderma multifidum* cultures (**B**,**D**).

The highest activity of proteolytic enzymes in the cultures of the fungal strains was detected in the first week of cultivation (Figure 1). High activity of caseinolytic protease was already noted after 4 days of cultivation of the *Arthroderma multifidum* strains and after 8 days in the *A. tuberculatum* cultures. However, the activity of this enzyme was almost two-fold higher in the *A. multifidum* cultures compared with the *A. tuberculatum* strains. After this time, the proteolytic activity gradually declined before day 16 in the *A. tuberculatum* culture and decreased continuously in the *A. multifidum* cultures. The decline in the protease activity in the *A. multifidum* culture (Figure 1D) may have been associated with the fact that the non-keratinous proteins contained in chicken feathers were degraded first, whereas keratin is decomposed at a later stage. The “cascade-like” activity of enzymes involved in the biodegradation of chicken feathers was reported by Bohacz [5]. The value of the coefficient of determination for the proteolytic activity in the *A. tuberculatum* and *A. multifidum* cultures (R^2^ = 58.4 and 68.9%, respectively) indicated a fairly large differentiation of the proteolytic activity of the fungal strains (Figure 1), with significant differences between the zone 1 strains labeled III, IV, V, XVIII, and XIX and the other strains (Table 3). The activity of proteolytic enzymes in the cultures of *Bacillus licheniformis* and *Stenotrophomonas maltophila* bacteria was reported to have a growing tendency up to cultivation day 2, followed by a decrease in this activity [40]. However, similar to the activity of keratinase, the proteolytic activity was higher than in the present study. Particular attention to the involvement of *Fervidobacterium islandicum* AW-1 in the decomposition of recombinant keratin was paid by Jin et al. [41]. They found that *F. islandicum* AW-1 crude extracts exhibited distinct keratinolytic activity against casein and recombinant keratin as a substrate. This activity was linearly correlated with the concentration of soluble proteins and free amino acids. The results of the PCA analysis performed in this study revealed that the activity of the caseinolytic protease was not significantly positively correlated with the release of soluble proteins and peptides, which was in contrast with the keratinase activity in both *A. tuberculatum* and *A. multifidum* cultures. This may have been related to the fact that the concentration of soluble proteins and peptides increases significantly during the decomposition of native keratin but not other non-keratin proteins. As reported by Kunert [24], proper fungal keratinolysis begins after the denaturation of feather keratin in the process of sulfitolysis.

The process of decomposition of native keratin by the analyzed fungal strains was accompanied by an increase in keratinase activity (exokeratinase). As indicated by Bohacz [5], this enzyme exhibited the maximum activity at the end of the experiment on *Aphanoascus fulvecens* fungi, i.e., around 45 days of cultivation. It was shown in the present study that the keratinase activity increased while the protease activity was low (Figure 1). This was confirmed by the mechanism of keratin decomposition by fungi from the *Chrysosporium* group. As reported by Kunert [24], the first step of keratinolysis consists of the biodegradation of simple proteins, followed by the decomposition of keratin proteins at a later stage. This was particularly evident in the cultures of the *A. tuberculatum* strains. The activity of this enzyme was similar to the activity of keratinase secreted by *Aspergillus parasiticus* [42]. The curvilinear regression analysis (R^2^ = 33.99 and 23.23%, respectively) revealed large differences in the keratinase activity between the investigated fungal strains. The statistical analysis of the homogeneous groups showed that the strains did not differ significantly from each other in the release of active keratinase, but they differed in the protease activity, in particular, for strains XVIII and XIX (Table 3).

### 3.4. Soluble Proteins and Peptides

**Figure 2:**
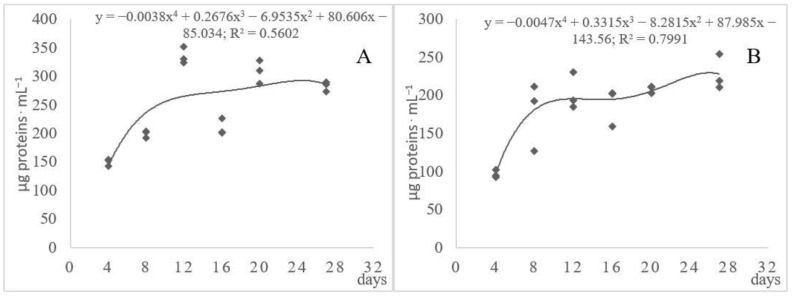
Dynamics of changes in the levels of soluble proteins and peptides in the cultures of *Arthroderma tuberculatum* (**A**) and *A. multifidum* (**B**) fungi involved in the decomposition of waste feathers.

As reported by Li et al. [39], after the reduction of disulfide bonds, feathers are hydrolyzed by proteases, which release peptides and free amino acids. The release of soluble proteins and peptides was noted in the *A. tuberculatum* and *A. multifidum* cultures containing native feather keratin as the sole source of C, N, S, and energy (Figure 2). An increase in the release of soluble proteins and peptides was noted in the first week of the culture of both fungi. The level of these substances increased with increasing cultivation time and was high on culture days 12 and 20–27. The concentration of these compounds in the cultures of the analyzed strains was 10-fold lower than in cultures of *Streptomyces* strains reported by Li et al. [39] and almost 2–3-fold higher than in *Arthroderma cuniculi* cultures [43]. The values of the coefficients of determination (R^2^ = 56.02 and 79.91%) indicated the low differentiation of the release of these substances between the cultured fungal strains. The statistical analysis of the homogeneous groups showed that strains V and VIII differed significantly from the other fungal strains (Table 3).

### 3.5. Ammonium and Sulfate Ions

**Figure 3:**
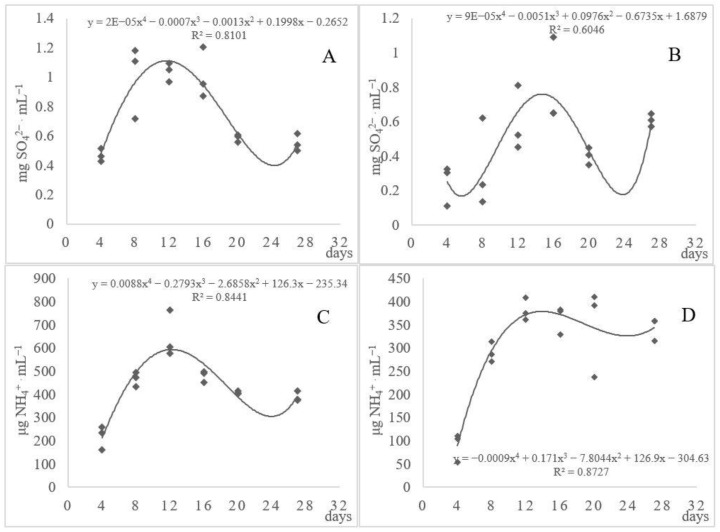
Dynamics of the changes in the levels of sulfate ions (**A**,**B**) and ammonium ions (**C**,**D**) in the cultures of *Arthroderma tuberculatum* (**A**,**C**) and *A. multifidum* (**B**,**D**) fungi involved in the decomposition of waste feathers.

As reported by Bohacz and Korniłłowicz-Kowalska [11], the narrow C/N ratio in feathers promotes the mineralization of nitrogen contained in feather keratin. The present experiment showed that *A. tuberculatum* accumulated 233.24–764.89 µg N-NH_4_^+^∙mL^−1^ in the substrate and *A. multifidum* accumulated 54.16–410.78 µg N-NH_4_^+^∙mL^−1^ (Figure 3). The most intensive deamination was recorded in the first two weeks of fungal growth on the feathers; afterward, the accumulation of ammonium ions declined. As demonstrated by Cavello et al. [44], ammonia was released during the first 6 days of hair keratin decomposition in *Paecilomyces lilacinus* fungal cultures. In the present study, strain III was considerably active in terms of N-keratin mineralization, whereas strains VIII and XIX were the weakest ammonificators and differed significantly from the other strains (Table 3).

Due to the high sulfur content in feather keratin, it was reasonable to determine the basic products of the mineralization of this component. As shown by Kunert [24], the decomposition of keratin by dermatophytes and fungi of the genus *Arthroderma* is accompanied by the fungal release of inorganic sulfite, in the presence of which, the disulfide bonds of keratin are cleaved into cysteine and sulfocysteine. The present results indicated the peak of organic S mineralization between days 8 and 12 of the *Arthroderma tuberculatum* culture and on day 16 in the case of the *A. multifidum* culture (Figure 3). The production of sulfates increased at the advanced stages of the proteolysis and ammonification processes. The sulfate release declined at the end of the experiment and increased again on day 27, especially in the *A. multifidum* cultures. Strains V and XVIII differed significantly from the other fungal strains in the release of sulfate ions. The cultures of strain V exhibited the highest concentration of these ions (Table 3). The coefficients of determination at the level of 60.0% and 80.0% indicated relatively low differentiation between the strains in the secretion of these mineral compounds.

### 3.6. Changes in pH

The present experiments showed (Table 4) that the degradation of native feathers by all analyzed *Arthroderma tuberculatum* and *Arthroderma multifidum* strains was accompanied by the alkalization of the culture medium. The highest pH values in the post-culture fluids were recorded on day 12 of the *A. tuberculatum* cultivation (pH = 8.76–8.79) and on days 16–20 of the *A. multifidum* culture (pH = 8.59–8.72). Afterward, the pH value decreased in both cultures. As demonstrated by Godheja and Shekhar [45], mold fungi of the genera *Trichoderma*, *Gliocladium*, *Fusarium*, *Syncephalastrum*, and *Aspergillus* contributed to the alkalization of the substrate (pH 8.0–9.0) during the feather decomposition process, with a maximum on cultivation day 20. The authors explained this phenomenon in terms of the deamination of the keratin substrate. The increase in pH was correlated with keratinase activity. The highest keratinolytic activity of *Paecilomyces marquandii* was detected by Gradišar et al. [46] at pH 8 and a temperature of ≈60 °C. The values of the coefficient of determination (R^2^ = 87.28 and 94.07% for the *A. tuberculatum* and *A. multifidum* cultures, respectively) indicated small changes in pH in the case of both strains, which was also confirmed by the statistical analysis of the homogeneous groups (Table 3).

### 3.7. Amino Acid Composition

Näshlom et al. [47] reported that the rate of amino acid production in some ecosystems is much higher than the production of mineral N forms. However, in some environments, they may be unavailable to plants due to immobilization by soil microorganisms. As suggested by these authors, amino acids can be absorbed by plants as a source of N. Keratin hydrolysates are a good source of amino acids [8,13,40]. The amino acid composition of the keratin hydrolysates from the *A. tuberculatum* cultures is presented in Table 5. The highest concentrations of tryptophan, cysteine, asparagine, glutamine, and serotonin were recorded in the tested hydrolysates, and these values ranged from 0.0356 to 0.0764 mg∙g^−1^. The values are different from those that were obtained by Dos Santos Cardoso et al. [48] in hydrolyzed feather protein and by Kumari and Kumar [49].

### 3.8. Principal Component Analysis

The main factors responsible for the biodegradation of feather keratin by the *Arthroderma tuberculatum* and *Arthroderma multifidum* strains were determined based on the adopted PCA analysis criteria.

**Figure 4:**
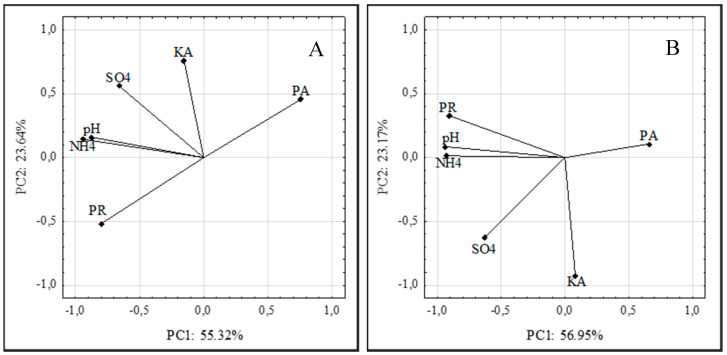
Plots of the variables. Locations of the load vectors toward two principal components for cultures of strains of *Arthroderma tuberculatum* (**A**) and *Arthroderma multifidum* (**B**) with feather waste; proteolytic activity (PA); keratinase activity (KA); soluble proteins and peptides (PR); ammonium ions (NH4); sulfate ions (SO4).

The analysis was based on an assessment of the enzymatic activity and the content of mineral products of keratin degradation in the fungal post-culture fluids with chicken feathers as the sole C, N, S, and energy source. It explained 78.96% and 80.05% of the variability using two principal components (PC1 and PC2) in the *A. tuberculatum* and *A. multifidum* cultures, respectively (Figure 4A,B). The first component (PC1) explained 55.32% and 56.95% of the data variability in the *A. tuberculatum* and *A. multifidum* cultures, respectively.

During the process of feather biodegradation by the *A. tuberculatum* strains, PC1 was significantly negatively correlated with protease activity (88.78%) and positively correlated with soluble proteins, peptides, and ammonium ions (95.35% and 73.22%, respectively). In the cultures of these fungi, PC2 explained 23.6% of the feather decomposition and correlated with the increase in the keratinase activity (73.12%) and sulfates (82.40%). During the process of feather biodegradation by the *A. multifidum* strains, PC1 was more significantly positively correlated with the pH of the post-culture fluids (93.88%), soluble proteins and peptides (94.10%), and ammonium ions (92.88%). In the cultures of these fungi, PC2 explained 23.17% of the feather decomposition and correlated with the increase in keratinase activity (91.54%).

## 4. Conclusions

The present data allowed for the conclusion that the biodegradation of feather keratin by all the analyzed *Arthroderma tuberculatum* and *Arthroderma multifidum* strains isolated from the soil that was directly influenced by the *Corvus frugilegus* rook colony (zone 1) and the soil from zone 4 (beyond the range of the colony impact) was primarily determined by the secretion of active proteases. This was associated with an increase in the level of soluble proteins and peptides and an increase in the concentration of ammonium ions accompanied by alkalization of the substrate. The keratinase activity was highly correlated with the release of sulfates, especially in the cultures of the *A. tuberculatum* strains. The *A. tuberculatum* strains isolated from the soil with the highest supply of organic matter, i.e., strains III, IV, and V, exhibited the lowest enzymatic activity compared to the *A. multifidum* strains, but they released mineral nitrogen and sulfur, which are important from a fertilization point of view, as well as nutritionally important peptides and amino acids.

## Figures and Tables

**Table 1:** Results of species identification based on PCR and nucleotide sequencing.

No.	Strains	Similarity (%)	Highest Sequence Similarity (%)
III	*Arthroderma tuberculatum*	99	100
IV	*Arthroderma tuberculatum*	99	100
V	*Arthroderma tuberculatum*	99	100
VIII	*Arthroderma multifdum*	98	100
XVIII	*Arthroderma multifdum*	98	100
XIX	*Arthroderma multifdum*	98	100

**Table 2:** Loss of waste feather weight that was induced by the activities of *A. tuberculatum* and *A. multifidum.*

Strains	Loss of Waste Feather Weight (%)
III	39.83 ^b^ *±* 0.025
IV	39.30 ^b^ *±* 0.007
V	43.00 ^b^ *±* 0.036
VIII	30.03 ^a^ *±* 0.077
XVIII	24.00 ^a^ *±* 0.006
XIX	27.00 ^a^ *±* 0.033

Explanation: The same letters (a, b) denote the means that form homogeneous groups. Therefore, when two compared means were assigned the same letter (e.g., a and a), they did not differ significantly (at the significance level α = 0.05). On the other hand, when a compared pair of means was assigned different letters (e.g., a and b), these means differed significantly (at the same significance level α = 0.05).

**Table 3:** Keratinolytic activity and release of mineral and organic forms of nitrogen and sulfur in different strains.

Strains	Protease	Keratinase	Soluble Proteins and Peptides	pH	N-NH_4_^+^	S-SO_4_^2−^
	(μg tyrosine mg^−1^ of protein)	(U mg^−1^ of protein)	(mg proteins mL^−1^)		(μg NH_4_^+^ mL^−1^)	(mg SO_4_^2−^ mL^−1^)
III	254.34 ^a^ ± 105.963	18.84 ^a^ ± 8.248	238.21 ^ab^ ± 60.620	8.55 ^a^ ± 0.197	461.13 ^b^ ± 160.125	0.71 ^abc^ ± 0.236
IV	236.97 ^a^ ± 100.289	16.65 ^a^ ± 7.972	245.46 ^ab^ ± 68.769	8.47 ^a^ ± 0.343	413.23 ^ab^ ± 135.437	0.75 ^bc^ ± 0.286
V	240.39 ^a^ ± 134.957	19.05 ^a^ ± 8.558	259.06 ^b^ ± 70.818	8.51 ^a^ ± 0.217	431.15 ^ab^ ± 105.577	0.86 ^c^ ± 0.314
VIII	470.18 ^ab^ ± 263.830	23.24 ^a^ ± 10.314	173.48 ^a^ ± 50.876	8.09 ^a^ ± 0.659	285.07 ^a^ ± 120.855	0.60 ^abc^ ± 0.324
XVIII	620.53 ^b^ ± 288.580	23.60 ^a^ ± 6.228	187.02 ^ab^ ± 40.337	8.36 ^a^ ± 0.535	321.41 ^ab^ ± 97.693	0.43 ^a^ ± 0.183
XIX	647.57 ^b^ ± 306.959	21.47 ^a^ ± 3.332	189.64 ^ab^ ± 49.001	8.35 ^a^ ± 0.546	301.29 ^a^ ± 96.615	0.46 ^ab^ ± 0.162

Explanation: The same letters (a, b, c) denote the means that form homogeneous groups. Therefore, when two compared means were assigned the same letter (e.g., a and a), they did not differ significantly (at the significance level α = 0.05). On the other hand, when a compared pair of means was assigned different letters (e.g., a and b), these means differed significantly (at the same significance level α = 0.05).

**Table 4:** Changes in the pH of the post-culture liquids of *Arthroderma tuberculatum* and *Arthroderma multifidum* isolated from soil.

Strains	Days of Culturing					
	4	8	12	16	20	27
III	8.21 ± 0.04	8.69 ± 0.08	8.79 ± 0.04	8.47 ± 0.03	8.50 ± 0.07	8.63 ± 0.06
IV	7.76 ± 0.17	8.64 ± 0.05	8.77 ± 0.03	8.51 ± 0.03	8.48 ± 0.06	8.66 ± 0.04
V	8.12 ± 0.01	8.73 ± 0.04	8.76 ± 0.03	8.464 ± 0.01	8.41 ± 0.03	8.58 ± 0.02
VIII	6.67 ± 0.19	8.06 ± 0.13	8.47 ± 0.01	8.38 ± 0.03	8.59 ± 0.02	8.56 ± 0.01
XVIII	7.20 ± 0.01	8.35 ± 0.01	8.64 ± 0.00	8.72 ± 0.01	8.68 ± 0.02	8.58 ± 0.02
XIX	7.17 ± 0.03	8.26 ± 0.01	8.63 ± 0.02	8.72 ± 0.01	8.71 ± 0.01	8.58 ± 0.00

**Table 5:** Amino acid composition (mg∙g^−1^) of keratin hydrolysates in the *A. tuberculatum* culture (III).

**Asp**	**Thr**	**Ser**	**Glu**	**Pro**	**Gly**	**Ala**	**Cyst Acid**	**Sulf Met**
0.0456	0.0113	0.0356	0.0410	0.0027	0.0192	0.0065	0.0648	0.0154
**Val**	**Ile**	**Leu**	**Tyr**	**Phe**	**His**	**Lys**	**Arg**	**Trp**
0.0166	0.0024	0.0074	0.0046	0.0057	0.0156	0.0139	0.0080	0.0764
